# The mirrored burden and accountable handovers in cancer survivorship: co‑designing a post‑treatment transition standard

**DOI:** 10.1038/s44401-026-00125-9

**Published:** 2026-08-05

**Authors:** Patrick Kierkegaard, Bowen Su, Dorothy Gujral, Karen Liesching-Schroder, Pernilla Lagergren, Alison McGregor, Justin Grayer

**Affiliations:** 1 Cancer Research UK Convergence Science Centre, Imperial College London and the Institute of Cancer Research, London, UK; 2 Department of Surgery and Cancer, Imperial College London, London, UK; 3 Department of Radiotherapy, Imperial College Healthcare NHS Trust, Charing Cross Hospital, London, UK; 4 Patient and Public Involvement & Engagement Representative, Mouth Cancer Foundation, Surrey, UK; 5 Department of Molecular Medicine and Surgery, Karolinska Institutet, Stockholm, Sweden; 6 Adult Psychological Support Service, The Royal Marsden Hospital, London, UK

**Keywords:** Cancer, Gastroenterology, Health care, Oncology

## Abstract

Post-treatment transitions after active cancer care often lack clear ownership, timelines and closure rules, shifting coordination work onto survivors and staff. We aimed to identify how these failures arise and to co-design a handover standard for post-treatment survivorship care. In a two-stage qualitative study, Stage A involved semi-structured interviews with survivors and professionals (*n* = 65) across head and neck, colorectal and gastrointestinal stromal tumour pathways; Stage B comprised four co-design workshops (*n* = 21). We identified a mechanism we term mirrored burden, in which routine letters and electronic records fail to make responsibility explicit and both survivors and staff end up chasing care. Co-design produced a four-component handover standard centred on accountable correspondence, a clinically senior survivorship lead practitioner with delegated authority, clinic-based resolution capacity, and equity safeguards for non-digital access and communication needs, plus four equity-stratified audit-ready feasibility indicators for service-level evaluation.

## Introduction

Transitions at the completion of specialist treatment remain a recurrent weak point in health systems. Across long term conditions, poorly specified handovers between specialist and community services are linked to loss to follow up, delay in acting on test results, unplanned emergency use and avoidable anxiety. These are socio‑technical coordination problems as they depend on how information, authority and tasks are configured across services and records, rather than on any single clinician’s behaviour^1,2^. Cancer survivorship illustrates this clearly. Worldwide, more than 50 million people are alive within five years of a cancer diagnosis, many with persistent late effects that require coordinated follow up across oncology, primary care and supportive services^3,4^. Survivors frequently report chronic pain, fatigue, functional limitations and unmet supportive care needs that depend on reliable cross team action rather than single discipline care^5–11^. For head and neck cancer, about half of survivors report at least one moderate or high unmet supportive care need and around one third live with persistent pain^12,13^.

International policy now mandates integrated survivorship care^14–17^, often shifting activity towards primary care, digital health and patient initiated follow up (PIFU)^18,19^. In parallel, national standards emphasise high quality communication and parity for non-digital access routes^20,21^. Yet the transition from treatment to survivorship remains a high risk interface. In many services, the next owner, action, due by date and closure rule are not stated in routine correspondence or records. Survivors and clinicians describe chasing results, appointments and responses across organisational boundaries, generating delay and patterned inequities rather than reliable planned follow up^22–28^. Survivorship care studies show consistent reports of fragmented communication, unclear division of responsibility and difficulty navigating post treatment care despite the existence of survivorship guidelines^29–31^. In health‑information‑technology terms, these failures reflect how ownership, timelines and closure are (or are not) encoded and surfaced in electronic patient records and routine correspondence ^32–34^.

When routine systems do not show who owns the next step, survivors and staff both end up chasing appointments, results and responses to keep care moving. We use the term mirrored burden for this health-system mechanism: coordination work is displaced onto both groups because accountability signals are not visible in routine correspondence and records. This mechanism extends Burden of Treatment theory^35^, which focuses on the balance between workload and patient capacity, and work on patient work^36^, by specifying the shared pattern of unpaid coordination labour across survivors and staff. It also connects with the administrative burden literature by locating these shared costs in opaque coordination infrastructure rather than in individual behaviour^37^. Mirrored burden is socially patterned: those with less time, less secure employment, more complex lives or fewer resources are less able to keep returning until someone acts.

Current approaches have not closed this accountability gap. Across survivorship models, quality of life and clinical outcomes seldom differ according to whether follow up is led by specialists, nurses, allied health professionals or primary care clinicians^38^. Survivorship care plans (SCPs) improve understanding and some aspects of communication^39,40^, but do not guarantee timely cross team action and are less often received by survivors with multiple long term conditions or disabilities^25,26^. PIFU has not shown survival advantages; qualitative syntheses indicate that it requires clear risk stratification, self-management support and rapid re-entry routes and may be unsuitable for people with high anxiety, low health literacy or psychosocial vulnerability^41–43^. Effects on coordination are likely to depend on visible accountability for actions rather than on information provision or nominal role allocation alone.

We therefore sought to design a practical, auditable health‑system handover standard for post‑treatment transitions. Our objective was to specify a minimal set of socio‑technical requirements that would convert survivorship correspondence and electronic patient‑record entries into trackable tasks with explicit ownership, timelines and closure rules, and to embed equity safeguards so that non‑digital routes do not become second class. We conducted a two‑stage qualitative study to: (1) diagnose accountability failures at completion of active cancer treatment across contrasting tumour groups; and (2) co‑design a four‑component post‑treatment handover standard with equity‑visible, audit‑ready indicators suitable for feasibility evaluation in routine services.

## Results

### Participant characteristics

We conducted 65 interviews (38 survivors; 27 professionals) in Stage-A and four workshops with 21 returning participants (13 survivors; 8 professionals) in Stage-B (Table 1). Stage-A: Diagnostic Analysis.

**Table 1:** Participant characteristics across study stages

Characteristic	Category	Number (*n*)	%
**SECTION A: stage-A survivors** (***N*** = **38**)			
Tumour group	Head and Neck	20	53
	Gastrointestinal stromal tumour	12	32
	Colorectal	6	16
Time since treatment	6–11 months	8	22
	1–3 years	11	30
	3–5 years	7	19
	5–10 years	5	13
	>10 years	7	16
Age at interview	20–29	2	5
	30–39	7	18
	40–49	6	16
	50–59	10	26
	60–69	6	16
	70–79	5	13
	Not specified	2	5
Gender	Male	17	45
	Female	20	53
	Non-binary	1	3
Ethnicity	White	35	92
	Minority ethnic groups	3	8
Deprivation (IMD quintile)	1 (most deprived)	3	8
	2	7	18
	3	6	16
	4	9	24
	5 (least deprived)	7	18
	Not specified (no postcode)	6	16
NHS work experience	Yes	4	11
	No	34	89
Occupation category	Professional/Managerial	17	45
	Healthcare/Clinical	4	11
	Manual/Skilled	7	18
	Other (retired, self-employed, etc.)	10	26
Employment trajectory post-treatment	No vocational impact	17	45
	Vocational disruption	18	47
	Career re-evaluation	2	5
	Not specified	1	3
**SECTION B: Stage-A professionals** (***N*** = **27**)			
Professional group	Medical clinicians (surgeons & oncologists)	5	19
	Clinical Nurse Specialists	5	19
	Allied health professionals (SLT; Dietetics)	10	37
	Psycho-oncology	2	7
	Third-sector and navigators	5	19
Age range	20–29	2	7
	30–39	7	26
	40–49	12	44
	50–59	6	22
Gender	Female	23	85
	Male	4	15
Ethnicity	White	23	85
	Ethnic minority groups	4	15
**SECTION C: Stage-B wWorkshops** (***N*** = **21**)			
Survivors by tumour group (*N* = 13)	Head and Neck	8	61.5
	Colorectal	2	15.4
	GIST	3	23.1
Professionals by role (*N* = *8*)	Clinical Nurse Specialist	1	12.5
	AHP - Speech and language therapy	3	37.5
	AHP - Dietetics	4	50.0
All	Total Stage-B participants	21	100.0

Percentages use item-level denominators within each block, and not specified rows are retained for transparency. Small cells for ethnicity are aggregated as ‘Minority ethnic groups’ (*n* < 5) to protect confidentiality. Values are descriptive only: no inferential or multiplicity adjustments are applied. Percentages may not sum to 100 due to rounding.

*AHP* allied health professional, *SLT* speech and language therapy, *IMD* index of multiple deprivation.

**Fig. 1:**
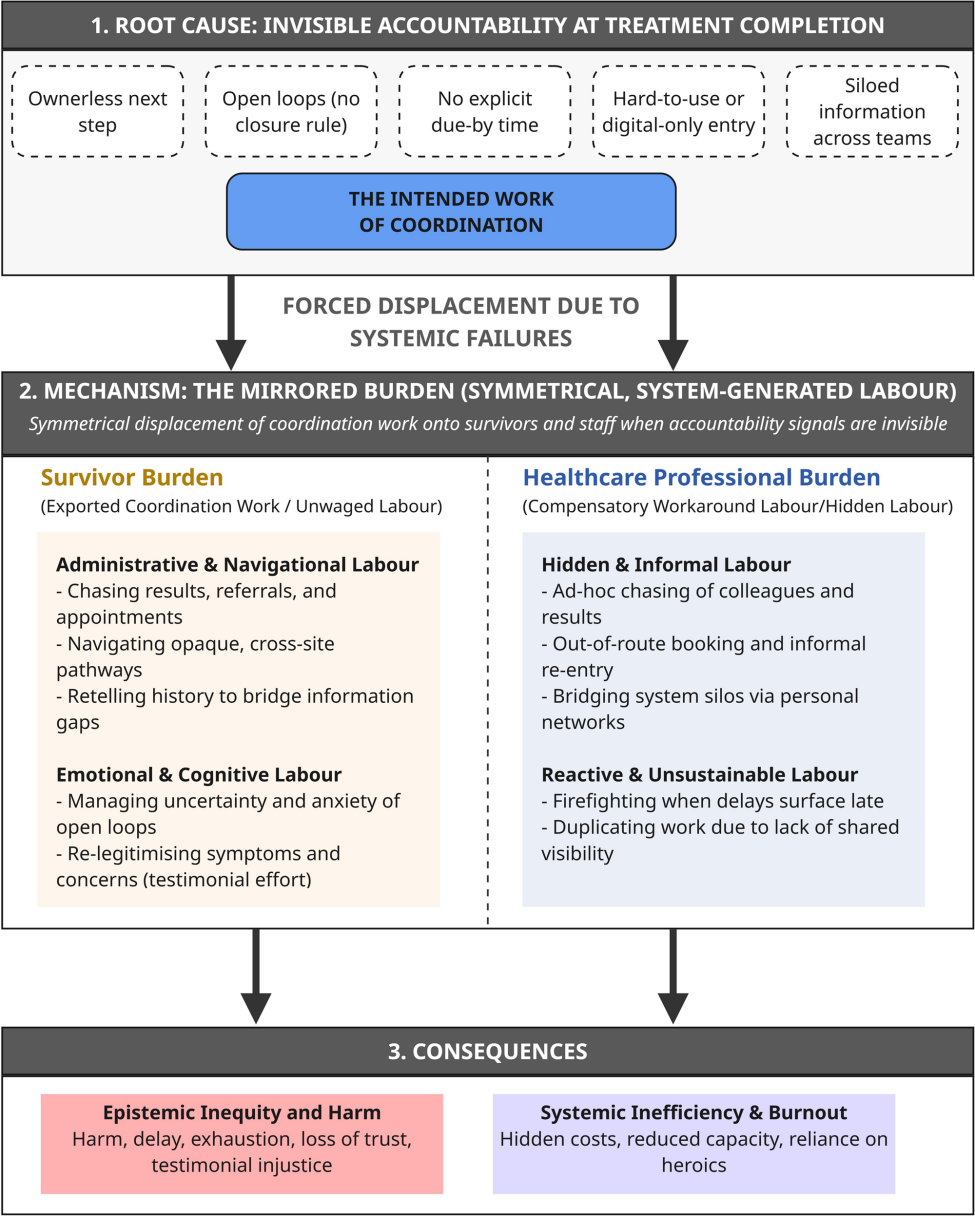
Mirrored burden mechanism at completion of cancer treatment/ (1) *Root cause*: At the end of active cancer treatment, accountability for next actions is often invisible in fragmented systems, displacing the intended work of coordination. (2) *Mechanism*: This opacity forces a mirrored displacement of coordination work onto survivors and staff. Survivors assume unwaged administrative, navigational and emotional labour. Healthcare professionals mirror this with hidden workaround labour and reactive firefighting to achieve closure across organisational boundaries. (3) *Consequences*: The mirrored burden generates epistemic inequity and harm for survivors, and systemic inefficiency, hidden costs and burnout for services.

Across tumour groups and professional roles, interviews converged on a single dominant system mechanism: a ‘mirrored burden’ of coordination work. When accountability for post‑treatment actions is not visible, survivors and staff both take on hidden labour to chase results, track appointments and navigate fragmented services, improvising workarounds in place of reliable handovers (Fig. 1 and Table S1). The themes below describe how this mechanism is produced across routine transitions.

### Theme 1: Cliff‑edge discharge and loss of named ownership

Survivors described completion of active treatment as a cliff edge where visible ownership disappears: what happens next, who is responsible and by when were frequently opaque, leaving people unsure how to re‑enter care:

“You literally feel like you’ve fallen off a cliff and it’s sort of like off you go… there’s this sort of gap of time where you’re like, ‘Oh my God, there’s nothing happening’ and that takes quite a lot to get your head round.” (P[6]‑CRC‑Survivor).

Professionals recognised the same vacuum:

“The patients [are] becoming overwhelmed with: ‘who do I call? […] it’s that lack of process on the patient side, and then also on the MDT side, where they just may not get told the right person to call or that communication might not go through to the correct person.” (HCP[03]‑AHP).

This theme locates the primary failure not in individual information deficits but in the absence of explicit ownership and timelines in routine systems, creating an empty space into which the mirrored burden of coordination work begins to grow.

### Theme 2: Parallel coordination work (“patient as case manager”)

In the face of fragmented systems, survivors took on de facto case management roles, while staff described informal workarounds to move people through services, creating parallel coordination tracks outside formal pathways.

Survivors often undertook coordination tasks themselves while staff described discretionary workarounds:

“You really have to push and you have to become your own case manager…. because there isn’t anybody else. You are the one making the phone calls, writing the letters, chasing appointments, chasing results, and so on.” (P[29]‑HNC‑Survivor).

Staff mirrored this with discretionary workarounds to support patients requesting post-treatment support if formal processes stalled:

“We would bend the rules by telling the patients to go directly to A&E or taking them to A&E ourselves and sort of providing them with the information that we know will be the buzzwords to get admitted onto the ward or contacting [staff] we’ve got a good relationship with…” (HCP[05]‑AHP).

These accounts show how poor task-workflow fit and lack of shared visibility generate unwaged survivor labour and compensatory staff labour in parallel, embodying the mirrored burden mechanism at the operational level.

### Theme 3: Testimonial effort and epistemic inequity

Survivors repeatedly distinguished between being heard and being helped, describing sustained effort to establish that post‑treatment symptoms warranted action, while clinicians recognised that those who were less confident, less fluent or more deferential were at particular risk of under‑action.

‘They kept telling me it was normal. This is part of your healing journey. I’ve obviously never had a gastrectomy before, so it wasn’t until it became like really bad, I thought this isn’t part of my journey’. (P[21]‑GIST‑Survivor).

HCPs recognised under‑reporting and the need for active prompting:

‘They often don’t want to moan about their symptoms because they often feel like that, they’re being ungrateful. When you prompt them, they’re more willing to come forward’ (HCP[13]-Consultant).

This recurrent testimonial effort reframes unreliable follow up as an epistemic inequity: in an opaque system, action is most readily secured by those who can keep returning and insisting, further amplifying the mirrored burden for survivors with fewer resources.

### Theme 4: Interface and preparation gap

Boundary failures between organisations and variable, often purely verbal handovers meant that treatment plans, monitoring schedules and responsibilities changed across sites without being recorded in ways that others could implement.

‘I moved around quite a lot, so I feel like I got a bit of lost between two hospitals. I didn’t have scan or anything for like for a year… I have [to have] 12 injections every 8-to-12 weeks for the rest of my life. What happened was [that] I ended up not having any of those for like a long time, and I got quite unwell…’ (P[8]‑GIST‑Survivor).

This shows cross‑site interface failure when ownership and timelines are not explicit. Professionals reported that:

‘You’re creating a sort of ‘lost tribe’ of patients that constantly bounce back and forth between these two [primary care and secondary care]. Healthcare is sort of pseudo-constructs. No-one’s really got responsibility or ownership of the problem because those problems are multidisciplinary’. (HCP[27]‑Consultant).

These interface gaps illustrate how weak cross‑service ties and inconsistent preparation convert nominal pathways into fragile chains of contingent workarounds, again displacing coordination tasks onto survivors and frontline staff.

### Theme 5: Equity‑relevant access barriers

Access to acknowledgement and re‑entry was patterned by communication and digital infrastructure: some survivors needed first‑class non‑digital routes (phone, letter, relay) and durable communication‑need flags to make contact safe and reliable.

‘One of the challenges I have is that every time I hear from them, I say, please do not phone me. Please set up a visual crew or I can attend in-person’. (P[19]‑HNC‑Survivor).

Clinicians echoed the concern regarding digital exclusion:

‘There’s a still a high percentage [older population] that really struggle with that technology and we have a lot of people come in the Centre saying, ‘where’s the message function on my app?’ Or why don’t I get a letter anymore?’ (HCP[24]‑ Third‑sector).

These accounts show how default digital pathways and fragile recording of communication needs risk making acknowledgment and re‑entry least reliable for those with sensory, cognitive or social barriers, reinforcing the socially patterned nature of the mirrored burden.

### Stage B: From diagnosis to workable design

Stage-B treated the five Stage-A mechanisms as design constraints for a post‑treatment handover standard. Workshops used these mechanisms to structure agenda priorities (Table S2): survivors specified the service functions required to remove ‘chase work’ (Patient Charter, Table S3), and professionals appraised workability, governance and scope for a clinic‑based role (Table S4). We treated the resulting specification as a socio‑technical response to system‑level failures in post‑treatment transitions.

### Survivors workshop: the patient charter

Survivors specified three requirements that map directly to the post‑treatment handover standard: relational accountability, infrastructural accountability and designed equity.

#### Relational accountability

Survivors required a clinically credible, reachable anchor with expertise in late effects, authorised to triage, book across services and escalate, thereby eliminating survivor “chase work” and clarifying who owns the next step:

‘I’d like to see a dedicated point of contact, rather than having to jump through hoops quite often… There’s guys [here] that have got speech issues quite often. I’m having to go through PALS (Patient Advice and Liaison Service) to chase an appointment or something because I’m nonverbal that day…’ (WS[09]‑HNC).

They emphasised that this anchor must have sufficient authority to challenge and navigate institutional hierarchies:

‘They should be the people that are brave enough to stand up to a consultant and say, ‘Excuse me, [patient X] came in mentioned something to you. You haven’t addressed his concern, or it’s clear he’s having a problem’’ (WS[03]‑HNC).

#### Infrastructural accountability

Survivors prioritised a short, actionable Care Transition Plan (what, by whom, by when) and closed‑loop correspondence that makes ownership and timelines universally visible. They also required durable signals in the record so that history and needs are surfaced before encounters:

‘I think it’d be useful if the tool could give you some feedback, almost like a tracking so if you order something online… Where you know where your query is or what that feedback is rather than thinking, ‘I’ve raised a concern or problem, where is that and is someone looking at it? Or have I been lost in the system’’ (WS[15]‑HNC)

Survivors gave concrete re‑entry specifications, emphasising direct access without having to re‑enter through general routes:

‘I think we should have an ongoing tool for referring yourself for life. For example, I’ve got one with MaxFax. I don’t have to ring. I don’t have to get the GP to refer. I’ve got a text that I just click on, and it sends a thing to them, saying quickly what the problem is, and I need to be seen… There’s no such thing to do that for ENT or Oncology’. (WS[08]‑HNC).

#### Designed equity

Survivors were clear that digital cannot be the only door to access care, and that systems must accommodate communication and mobility constraints:

‘There’s a lot of patients out there that haven’t got family around them… They’re not able to navigate things or technology. [They] even struggle with getting out of their house, especially with the mental health aspect…’ (WS[11]‑HNC).

They also wanted a trusted channel under clinical oversight to avoid unsafe advice and provide supervised reassurance for non‑acute but worrying issues:

‘Is this normal? Should I be worried? I was fine and then I got a lump on my tongue, and it started burning… There’s nobody to ask these questions because it’s not an acute problem. It’s a long-term effect’. (WS[08]‑HNC)

Taken together, the Charter translates the mirrored burden into system requirements: a named anchor with real authority, infrastructure that makes tasks and timelines visible and trackable, and equity safeguards built into access routes.

### Professional workshops: appraising workability

Clinicians translated the Charter into a clinic‑based Survivorship Lead Practitioner (SLP) role. They defined this as a single clinical front door with authority to triage, book across services, assign default results ownership and escalate on timers from a clinic base. They stressed that the SLP must formalise and resource existing clinical expertise rather than creating an administrative helpline, and that mandated authority is necessary to ensure action:

‘The CNSs I’ve worked with are already doing this job and are outstanding and the patients would go to them with any of these kinds of concerns…. I think this could be a CNS job, although I know CNSs are incredibly busy already’ (WS[15]‑AHP).

Seniority and scope were central to legitimacy and safety. Clinicians argued that the role must be experienced enough to judge ‘normal’ versus ‘needs action’ across pathways:

‘I almost think Band-6 [mid-grade] is too inexperienced… you’re not necessarily going to have seen enough patients over a long enough period of time to understand what symptoms are normal and what… actually needs a referral back to ENT’. (WS[03]‑AHP).

Workability hinged on clear downstream ownership and capacity when the SLP hands over the patient:

‘We’re only funded as a team up until the end point of the treatment. Who picks up the late effects? Who picks up the patient who’s now presenting two or three years later but doesn’t actually have an oncological problem that requires treatment?’ (WS[03]‑AHP).

Healthcare professionals emphasised resourcing and governance, arguing for a new, explicitly funded post rather than repurposing existing roles, and for clear measurement and sponsorship. They were also alert to balance, stating that the SLP should not crowd out self‑management or peer support:

‘We need to be very careful that that it doesn’t de-skill patients. Patients can be very resourceful in themselves, and I think developing a role that helps people develop self-care as well is really important’. (WS[14]‑CNS).

The professional workshops confirmed that the proposed SLP is feasible only if specified as a senior, clinic‑based transition lead with formal authority, protected time and explicit downstream agreements, rather than an informal, unfunded extension of existing roles.

### Post‑treatment handover standard: Specification and Workflow

Synthesising the Patient Charter and professional workability appraisal, we specified a four‑component post‑treatment handover standard. The core is the OADC rule set for routine correspondence (letters and notes), with two‑way acknowledgement, identical content for survivors and clinicians, and task‑status visibility.

**Fig. 2:**
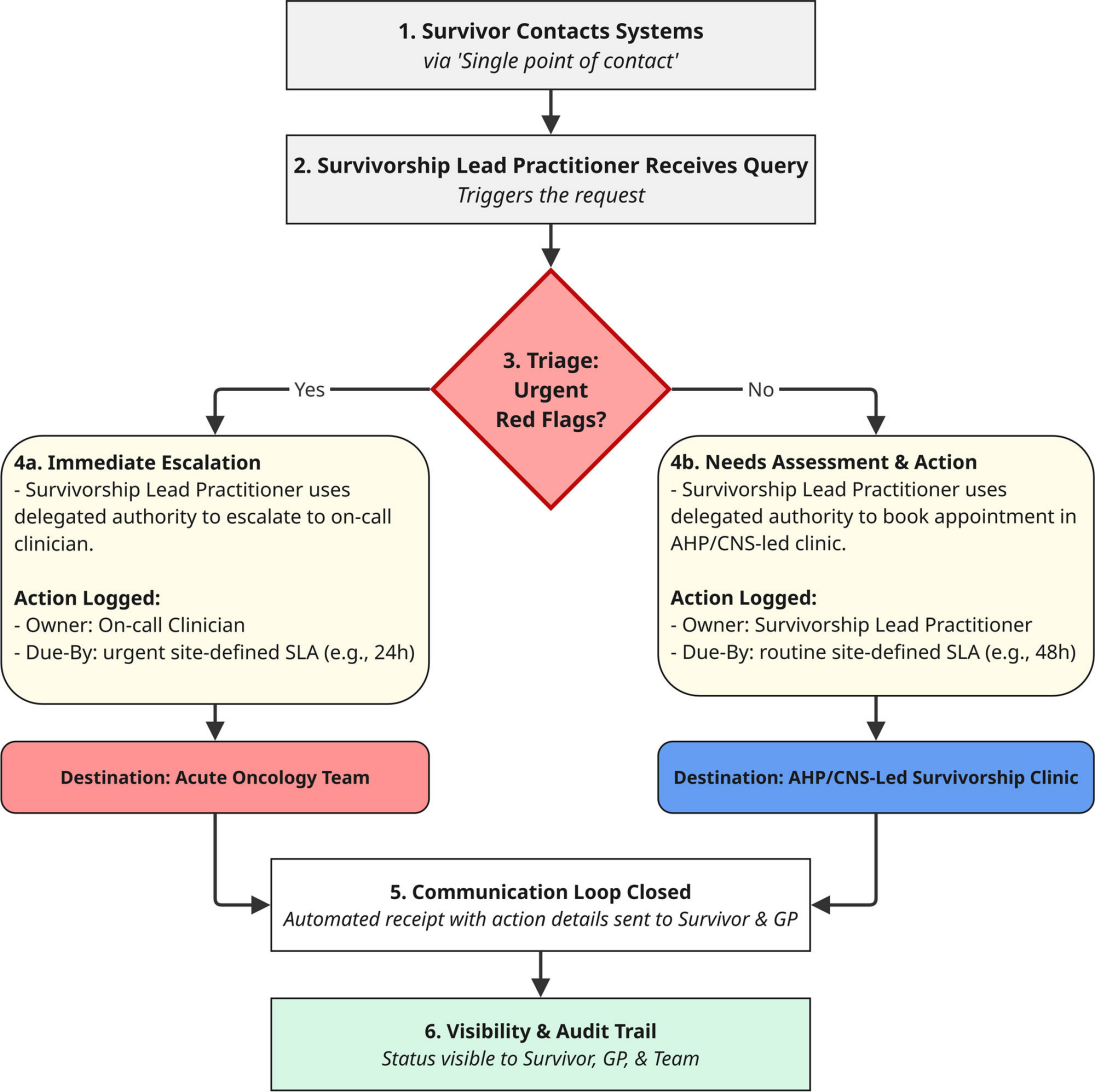
Operational workflow for the post‑treatment handover standard. The diagram shows the Survivorship Lead Practitioner (SLP) workflow from survivor contact to acknowledgement and closure under time‑bound rules. Step 1 is survivor contact through a single front door. Step 2 routes the request to the clinic‑based SLP, who records Owner, Action, Due‑by and Closure (OADC) in the record. Step 3 shows triage for urgent red‑flag symptoms. Non‑urgent requests proceed to needs assessment and booking in the allied health professional or clinical nurse specialist‑led survivorship clinic (step 4b); urgent requests are escalated directly to the acute oncology team (step 4a). Step 5 closes the loop with an automated receipt and action summary to the survivor and general practitioner. Step 6 makes item status visible to the survivor and teams through a shared audit trail. The workflow aligns with the minimal specification in Table 2 and the indicator definitions in Table S6. Timeframes shown in the workflow are illustrative examples of site-defined service-level agreements for future feasibility testing; exact thresholds are locally configurable and should be version-logged locally.

Alongside accountable correspondence, the standard comprised a clinically senior, clinic-based SLP with delegated authority for triage, cross-boundary booking and time-bound escalation, clinic-based resolution capacity, and equity safeguards. Table 2 summarises these four components and the four equity-stratified feasibility indicators specified for future evaluation. Figure 2 shows how the components work together in the operational handover workflow, and Table 3 links the main Stage-A failure mechanisms to the corresponding Stage-B design features. The minimum conditions in Table 2 define the minimum floor for judging whether a component is in place in a future feasibility test; site-defined timeframes remain locally configurable and are detailed in the supplementary implementation materials. Full implementation specifications are provided in Tables S5–7 and Fig. S2 presents the implementation logic model.

**Table 2:** Minimal specification for the post‑treatment handover standard and linked feasibility indicators

Block A: Minimal specification set for future feasibility evaluation					
Component	What it is (operational specification)	Why this matters	Minimum conditions for implementation in future feasibility testing		Linked feasibility indicator(s)
1. Accountable correspondence	All survivorship‑relevant letters and electronic patient record (EPR) notes state Owner, Action, Due‑by, Closure (OADC); two‑way acknowledgements are recorded; identical content goes to the survivor and general practitioner; item status is visible (acknowledged / in progress / closed).	Invisible ownership shifts coordination work onto survivors (unwaged labour) and compels staff workarounds (compensatory labour). Identical content and status visibility are intended to reduce failure demand.	Every letter/note shows all four OADC fields; acknowledgements are recorded (manual audit log if read‑receipts are absent); status is visible across teams; identical content is sent to the survivor and involved healthcare professionals; staff have the agreed right to return non‑OADC items for completion.		Time‑to‑acknowledgement; loop‑closure within timeframe; results‑ownership compliance; channel‑concordance (all equity‑stratified).
2. Clinically senior SLP (delegated authority)	A senior clinical nurse specialist or allied health professional functions as a single clinical front door from a clinic base, with delegated triage, cross‑boundary booking and time‑bound escalation (to a duty clinician or multidisciplinary team).	Availability without authority created new queues and unresolved messages; combining authority with a clinic base converts requests into actions and closure, rather than messages that generate further work.	Seniority and scope are specified in standard operating procedures; job plans include protected clinic time; booking rights and escalation routes are in place; a reachable clinical front door exists (not a generic administrative helpline), with a directory of named contacts and response SLAs.		Loop‑closure within timeframe; time‑to‑acknowledgement.
3. Clinic-based resolution capacity	A staffed clinic base holds time and relationships so the Survivorship Lead Practitioner can act (book, order, escalate) rather than simply relaying messages.	Lone inboxes were unsafe and created parallel, unresourced queues; a clinic base enables real closure.	Named clinic base with rota and cover; live access to booking across services; explicit escalation routes; no parallel ‘shadow’ queue.		Loop‑closure within timeframe; results‑ownership compliance.
4. Equity safeguards	First‑class non‑digital routes (phone/letter/relay); durable communication‑need flags (Accessible Information Standard / SNOMED where available) propagate across services; equity‑visible reporting is produced each cycle.	Burden shifting was socially patterned; Parity rules for response times across digital and non‑digital routes are documented and monitored.	Preferred channel is recorded and honoured; durable flags are present in the record and correspondence; indicators are reported by subgroup with a missingness row.		Channel‑concordance, plus all indicators reported by equity‑relevant subgroup.
**Block B: Feasibility indicators (pre-specified feasibility indicator)**					
Feasibility indicator (co‑primary feasibility endpoint)	Operational definition	Unit	Numerator/Denominator	Missingness handling	Equity strata
Time-to-acknowledgement (I1)	Start: timestamp when a survivor or professional request reaches the service (logged call, message or letter); Stop: first recorded two‑way acknowledgement in the EPR or audit log.	Hours	Requests acknowledged within the site‑defined timeframe/all requests.	Record reason if start or stop is missing; record contact route.	Language; disability (AIS); ethnicity; Index of Multiple Deprivation quintile; age; tumour group.
Loop-closure within timeframe (I2)	Start: request logged/assigned. Stop: action completed and closure communicated (closure rule met).	Percent	Requests closed within timeframe/all actionable requests.	Code ‘no closure/unclear stop’ with reason.	As above
Results-ownership compliance (I3)	For each reportable result, a named owner, due‑by date, and closure note are visible to survivor and clinicians.	Percent	Results with all three fields present/all reportable results.	Code owner missing and other missing fields; retain in denominator.	As above
Channel-concordance (preferred route honoured) (I4)	Contact is made and acknowledged via the documented preferred channel; non‑digital routes have parity.	Percent	Interactions via the recorded preferred route/all interactions.	Record ‘preference unknown’; retain in denominator.	As above

Block A summarises the four components of the post-treatment handover standard, their rationale, and the minimum conditions used to judge whether each component is sufficiently in place for future feasibility evaluation, with the corresponding service-level indicators. Block B defines the four equity-stratified feasibility indicators (I1-I4), including units, numerators, denominators, missingness handling and equity strata. All four indicators are pre-specified feasibility measures for service-level reporting, not clinical outcomes.

All four indicators are pre-specified feasibility measures for service-level reporting. Any future analyses will be descriptive; the study is not powered or intended for effectiveness comparisons. Indicators are displayed as run or Statistical Process Control charts with equity stratification and a missingness row each cycle, with balance measures (for example, primary-care message volume and time-to-routine appointment) plotted in parallel. The ‘minimum conditions for implementation’ column defines the minimum floor for judging whether a component is in place in future feasibility testing. These conditions are intended to reduce under-specification, while site-defined timeframes and reporting thresholds remain locally configurable and version-logged. Full implementation conditions and expanded indicator definitions appear in Tables S5–6. No indicator data were collected in this co-design study.

**Table 3:** Diagnosis‑to‑design traceability for the post‑treatment handover standard

Mechanism (Stage-A) and CFIR 2.0 determinants	Minimal design feature (Stage-B) and NPT workability (operational signals)	Implementation strategies (ERIC); outcomes (Proctor); audit-ready indicators
*Cliff-edge discharge and loss of named ownership.* Abrupt end to treatment; no visible owner, action, due-by date or closure rule; impersonal letters instead of a structured handover. *CFIR*: Implementation process —planning; implementation process—engaging; inner setting—communications; outer setting—patient needs and resources.	*Accountable correspondence in letters/EPR notes* (OADC; two-way acknowledgements; identical content; cross-boundary booking; time-bound escalation). *NPT:* Coherence - Differentiation; Collective Action - Interactional workability; Reflexive Monitoring - Systematisation. *Operational signal:* OADC templates in routine use; right to return non-OADC items; status visible across oncology and primary care	*ERIC:* Develop a formal implementation blueprint; Change record systems; Conduct educational meetings; Conduct educational outreach visits; Obtain formal commitments; Audit and provide feedback. Outcomes: Acceptability; adoption; fidelity. *Indicators:* time-to-acknowledgement (I1); results-ownership compliance (I3).
*Parallel coordination work (‘patient as case manager’)*. Survivors chase letters and results across fragmented systems; staff ‘bend rules’ to push cases through when pathways stall. *CFIR***:** Inner setting— compatibility; inner setting— access to knowledge and information; inner setting— available resources; implementation process—doing.	*Clinic-based survivorship lead practitioner* as first clinical triage, with delegated authority, cross-service booking, and duty-clinician escalation. *NPT*: Coherence—differentiation; cognitive participation— enrolment/activation; collective action— contextual integration; reflexive monitoring—systematisation. *Operational signal***:** Protected clinic time; cross-boundary booking rights; named escalation routes.	*ERIC*: Revise professional roles; Create new clinical teams; Change record systems; Build a coalition; Organise clinician implementation team meetings; Provide ongoing consultation. *Outcomes*: Adoption; penetration; feasibility. *Indicator*: loop-closure within timeframe (I2).
*Testimonial effort and epistemic inequity*. Survivors must repeatedly re-legitimise symptoms to convert acknowledgement into action; access favours assertive, English-speaking survivors. *CFIR*: Inner setting—culture (learning-centredness; human-equality-centredness; recipient—centredness); individuals— capability; individuals— motivation; outer setting— local attitudes.	‘*Acknowledge→Act*’ rule in SOPs plus durable AIS communication-need flags; channel of choice honoured. *NPT*: Collective action—relational integration; cognitive participation— legitimation; reflexive monitoring— communal appraisal. *Operational signal*: Plain-language OADC in correspondence; visible communication-need flags that travel across letters and referrals.	*ERIC*: Identify and prepare champions; Tailor strategies; Use advisory boards and workgroups; Conduct local consensus discussions; Obtain formal commitments; Audit and provide feedback. *Outcomes*: Acceptability; appropriateness; equity; feasibility. *Indicators*: time-to-acknowledgement (equity-stratified) (I1); loop-closure within timeframe (equity-stratified) (I2).
*Interface and preparation gaps.* Boundary failures and verbal or variable plans; survivors left to ‘find’ information while recovering; inconsistent cross-site planning. *CFIR*: Outer setting—partnerships and connections; inner setting—communications; implementation process; implementation process - doing.	*Short Care Transition Plan* at treatment completion; first check-in booked; identical content to survivor and healthcare professionals; cross-boundary booking; time-bound escalation. *NPT***:** Coherence—communal specification; Collective action— interactional workability; Reflexive monitoring—systematisation. *Operational signal*: ‘What/by whom/by when’ fields completed; first check-in pre-booked.	*ERIC*: Develop a formal implementation blueprint; Develop educational materials; Facilitate relay of clinical data to providers; Develop resource sharing agreements; Conduct local consensus discussions; Conduct educational outreach visits. *Outcomes*: Fidelity; feasibility; sustainability. *Indicators*: Primarily: loop-closure within timeframe (I2); Secondarily: time-to-acknowledgement for post-CTP contacts (I1).
*Equity-relevant access barriers*. Digital and communication barriers make acknowledgement and re-entry unreliable; survivors request first-class non-digital routes. *CFIR***:** Outer setting—local conditions; outer setting— patient needs and resources; inner setting—information technology infrastructure; inner setting—access to knowledge and information; innovation— adaptability.	*Parity-by-design* for non-digital routes; durable AIS flags; channel of choice honoured. *NPT*: Collective action—contextual integration; reflexive monitoring— systematisation/reconfiguration. *Operational signal*: Preferred channel recorded and used; parity rules applied.	*ERIC*: Tailor strategies; change record systems; promote network weaving; prepare patients/consumers to be active participants; capture and share local knowledge. *Outcomes*: Appropriateness; Adoption; Feasibility. *Indicator*: Channel-concordance (equity-visible) (I4).
*Buffering factors (negative cases)*. Reachable named anchor and ‘open-door’ policy reduce anxiety and close loops when clearly explained. *CFIR***:** Inner setting—relational connections; inner setting— culture (recipient-centredness); implementation process— engaging; reflecting— evaluating.	*Corollary of the SLP and Care Transition Plan features*: Named, reachable anchor with authority and clear re-entry routes. *NPT*: Cognitive participation—legitimation; collective action— relational/interactional workability; reflexive monitoring—systematisation. Operational signal: ‘Open-door’ framed as patient-initiated follow-up with clinico-administrative closure rules.	*ERIC*: Revise professional roles; Build a coalition; Change record systems; Provide ongoing consultation. *Outcomes*: Acceptability; fidelity. *Indicators*: loop-closure within timeframe for re-entry contacts (I2); time-to-acknowledgement for contacts via the named anchor (I1).

Each row links a Stage‑A failure mechanism contributing to the mirrored burden to the corresponding Stage‑B minimal design feature, the relevant CFIR 2.0 determinants and Normalisation Process Theory workability signals, selected ERIC implementation strategies and Proctor implementation outcomes, and the aligned audit‑ready indicator(s). Rows are conceptual rather than statistical and are not subject to multiplicity correction. The full diagnosis‑to‑design lineage is provided in Table S7 and summarised in the Implementation Research Logic Model (Fig. S2).

Indicator key: I1 = time-to-acknowledgement; I2 = loop-closure within service timeframes; I3 = results-ownership compliance; I4 = channel-concordance (see Table 2 and Table S6 for full operational definitions). In planned feasibility evaluations, indicators will be reported as run or Statistical Process Control charts with equity stratification and a missingness row; site-specific thresholds will be version-logged. Indicators are pre-specified feasibility measures intended to test whether the standard can be applied reliably and equitably; no indicator data were collected in this co-design study. Representative survivor and healthcare professional quotations for each mechanism are provided in Table S1; full mirrored quotations, row-level dependencies, risks and balance measures are provided in Table S7. Box 1 provides one worked example of this diagnosis-to-design lineage in the main text.

Box 1. Worked example of diagnosis-to-design traceability

**Table:** 

Stage-A excerpt	Mechanism	Minimal design feature	Aligned feasibility indicator(s)
‘I’ve been through all that and then to just be released and told you’re going to go for scans every whatever six months, whatever it was. That’s it. Not really much more to it and it was probably just a letter as well that that told me that. It was kind of impersonal, I think’. (P[09]‑GIST‑Survivor).	*Cliff-edge discharge and loss of named ownership:* abrupt end to treatment; no visible owner, action, due-by date or closure rule; impersonal letters instead of a structured handover.	*Accountable correspondence in routine letters/EPR notes:* OADC (Owner, Action, Due-by, Closure) fields, two-way acknowledgements, identical content to survivor and GP, with cross-boundary booking and time-bound escalation.	**I1** time-to-acknowledgement; **I3** results-ownership compliance

Note: Full row-level diagnosis-to-design traceability for all mechanisms is provided in Table S7; paired survivor and professional exemplar quotations are provided in Table S1.

## Discussion

At completion of active cancer treatment, accountability for what happens next was often invisible. Survivors and staff described a mirrored burden of coordination work in which both groups chased results and appointments and relied on improvised workarounds to secure follow‑up. Using this diagnosis, we co‑designed a post‑treatment handover standard that makes task ownership, timelines and closure explicit in routine correspondence, locates triage and booking authority in a clinic‑based SLP, and embeds equity safeguards for non‑digital access and communication needs. We also specified four equity‑stratified feasibility indicators so that the standard can be evaluated as a service‑level, rather than individual‑level, intervention. Taken together, these elements position the handover standard as meso‑level health‑system infrastructure for high‑risk transitions rather than a tumour‑specific service innovation.

These findings characterise completion‑of‑treatment handovers as socio‑technical coordination problems generated by how letters, electronic patient‑record entries, clinic roles and escalation rules are configured across organisational boundaries, not by individual goodwill alone. Our use of the Consolidated Framework for Implementation Research, Normalisation Process Theory, ERIC strategies, Proctor’s implementation outcomes and an Implementation Research Logic Model makes this systems‑thinking lens explicit by linking mechanisms to minimal design features and audit‑ready indicators.

The mirrored burden mechanism extends existing theory by shifting attention from individual workload to system‑level accountability failures. Burden of Treatment theory and work on patient work describe the volume and organisation of tasks that patients must undertake relative to their capacity^35,36^, while administrative burden literature catalogues the learning, psychological and compliance costs imposed by opaque public systems^37^. Our analysis shows how, when letters and records do not state who owns a task, by when, and how closure will be recorded, those costs are displaced between survivors and staff rather than falling on patients alone. Survivors described repeated cycles of contacting services, explaining concerns and following up when nothing happened, while staff described bending rules and using personal networks to secure appointments or urgent review. This dyadic pattern is better understood as a property of the health system than of individual behaviour and corresponds to continuity and coordination failures highlighted in frameworks on integrated, people‑centred health services, which treat explicit responsibility and clear information flows across settings as core functions of resilient health systems ^44^.

These dynamics also have epistemic and distributional consequences. When action only follows repeated assertion, those who cannot keep returning or who are less likely to be believed face systematic disadvantage^45,46^. Work on epistemic injustice in healthcare underlines how credibility deficits can become structurally embedded, while survivorship studies show that stacked disadvantages in employment, income and housing already constrain people’s capacity to sustain engagement with care. In this context, mirrored burden is unlikely to be evenly distributed: those with the least reserve are asked to do the most coordination work ^47,48^.

Viewed through this mechanism, the modest outcome gains from many survivorship innovations are unsurprising. Across survivorship models, outcomes differ little according to whether follow‑up is led by oncology, nurses or primary‑care clinicians^38^. Survivorship care plans improve understanding and some aspects of communication but do not guarantee timely cross‑team action and are less often received by survivors living with multiple long‑term conditions or disabilities^25,40^. PIFU can reduce planned clinic visits in selected groups, but systematic reviews report no survival advantage and highlight concerns about suitability for people with high anxiety, low health literacy or complex psychosocial needs^41–43^. Reviews of survivorship care repeatedly describe fragmented communication, unclear responsibility and difficulty navigating services despite the presence of guidelines^29–31^. Recent survivorship‑care quality frameworks specify coordination and role clarity as core domains, but mostly as broad constructs rather than concrete task‑ownership and closure rules. The handover standard developed here can be read as a meso‑level operationalisation of those domains for high‑risk transitions^49,50^. In many settings, innovations change who sees whom and how often, or improve the information survivors receive, but leave the underlying accountability infrastructure untouched; when task ownership, timelines and closure rules remain implicit, mirrored burden persists ^51^.

The post-treatment handover standard is designed to make accountability visible and auditable at the point where survivorship care most often fragments. The OADC correspondence rule requires survivorship-relevant letters and electronic notes to state a named owner, a specific action, a due-by time and a closure rule, with two-way acknowledgement and shared status visibility, so that correspondence functions as a trackable task rather than a one-way communication. The clinic-based SLP complements this rule by providing a single, clinically credible front door with delegated authority to triage, book across services and escalate within agreed timeframes, rather than relying on informal courtesy or personal networks. Evidence on missed and delayed follow-up of test results in ambulatory care shows how unclear responsibility, timing and communication processes create patient-safety risks and require system-level solutions for clear ownership and follow-up^52^. These concerns are consistent with the Global Patient Safety Action Plan 2021-2030, which frames patient safety as a health-system priority across care settings ^53^.

Together, the OADC rule set and the clinic-based SLP re-specify both information and authority across services. This goes beyond patient-navigation programmes, which focus primarily on barrier removal and psychosocial support, and beyond traditional clinical nurse specialist roles, whose ability to act across organisational boundaries is often discretionary or relationship-dependent. By specifying formal authority for triage, cross-boundary booking, default results ownership and time-bound escalation, and by anchoring that authority in job plans and governance, the standard adapts closed-loop handover principles, akin to those in the I-PASS programme, to the post-treatment setting by standardising who is responsible, for what, and with what feedback ^54–57^. For survivors with complex late effects, persistent pain and unmet needs, a reachable and clinically credible triage point is therefore a safety requirement rather than a convenience, regardless of whether follow-up is specialist-led, shared care or patient-initiate^13,16,17,58,59^. The standard also aligns with person-centred survivorship policy and guidance on patient-facing correspondence by making responsibility explicit at the point of handover^20,60,61^. In commissioning terms, it is best understood as infrastructure for a safer transition rather than as a separate survivorship programme.

Equity was designed into the standard rather than added later. Survivors described acknowledgement and re-entry as least reliable when communication needs were poorly recorded and digital routes were treated as the default, echoing wider evidence that access to telemedicine in cancer care is patterned by socioeconomic disadvantage, geography, racial and ethnic inequity, and broadband availability and affordability^62,63^. Treating reliable non-digital access as a core design requirement is therefore consistent with broader commitments to equitable access to essential health services^64^. The standard accordingly treats non-digital routes as first-class rather than residual, with resourced phone, letter and relay channels and durable communication-need flags, including use of the Accessible Information Standard and SNOMED coding where available^21^. Channel concordance is monitored as a feasibility indicator, with equity-stratified reporting and a missingness row to make patterns of use and exclusion visible. Trauma-informed staff responses, including approaches such as #CheckWithMeFirst, may help survivors feel safer and more supported when seeking help for late effects ^65^.

The indicator set is intended to support feasibility evaluation and early warning, not to function as a surrogate for clinical outcomes. Time-to-acknowledgement, loop-closure within agreed timeframes, results-ownership compliance and channel concordance are specified as pre-specified feasibility indicators with explicit numerators, denominators, start-and-stop rules, equity strata and balance measures. They are designed to show whether services can apply the standard reliably and equitably before any claims are made about effects on readmissions, emergency use or quality of life. The “minimum conditions for implementation” should therefore be read as the minimum floor for judging whether a component is in place in a feasibility test, with site-defined timeframes and reporting thresholds subject to local agreement and version control. Run or Statistical Process Control charts with equity stratification and a missingness row are proposed as the main reporting format, consistent with the Making Data Count approach used in NHS trust board reporting^66^. This shifts attention from headline outcome targets to the reliability and fairness of the underlying coordination processes. System-level work on National Health Service administration describes similar recurring failures in appointments, test-result communication and knowing who to contact, and argues that administration should be treated as a core dimension of quality and productivity rather than background support^67^. The feasibility indicators proposed here offer one way to operationalise that argument for post‑treatment survivorship transitions.

Implementing this standard will require organisational authority and resources, not simply a relabelled role. An SLP expected to take on triage, booking and escalation work without protected time, cross-boundary booking rights or clear escalation routes would add to the hidden labour the standard is intended to reduce. Workshop participants were explicit that the role had to be senior, clinic-based and formally delegated, with job plans and governance aligned accordingly. Without these conditions, services risk new workarounds or superficial compliance, much as incomplete uptake of surgical safety checklists or persistent workarounds in electronic records emerges when systems do not fit clinical work^33,34,68–70^. The proposed implementation logic therefore emphasises revision of professional roles, coalition building across oncology, diagnostics, primary care and administration, and use of routine data to monitor and adjust the standard.

Although this work focused on cancer survivorship in two English specialist services, the coordination problems we identified are not inherently tumour‑specific. Similar combinations of unclear ownership, poor visibility of plans and reliance on workarounds have been described in other high-risk handovers across organisational boundaries. Transferability should therefore be understood at the level of function rather than form: explicit task ownership, timeframes, closure rules, cross-boundary booking and equity-sensitive access routes may be relevant in other specialist-to-community transitions, but the roles, thresholds and governance arrangements would require local adaptation and empirical testing.

This study has limitations that constrain interpretation. The survivor cohort was predominantly White, representation from socioeconomically deprived groups and the most deprived Index of Multiple Deprivation quintiles remained limited, and colorectal survivors remained under-represented. Because survivors were recruited through charities and an online expression-of-interest form, and Stage-B involved returning participants, the accounts and workshop priorities are also likely to over-represent people who were willing and able to engage repeatedly with research. The mirrored burden we describe may therefore be underestimated, and the co-designed standard may not yet fully reflect the needs of ethnically diverse, more deprived, or less digitally connected populations. Future feasibility and pilot work should therefore recruit beyond charity and online routes, retain non-digital participation options, and work through services and community partners that can better reach under-represented survivor groups.

Professional participants were drawn from two London‑based trusts and third‑sector partners and did not include primary‑care clinicians or commissioners, although the proposed standard spans interfaces with those sectors. Stage‑B workshops did not include senior budget holders, so resourcing decisions and opportunity costs were discussed but not negotiated. Finally, this was a co‑design and specification study: we did not collect indicator data or clinical outcomes, and we did not assess cost‑effectiveness. All claims are therefore restricted to mechanism, design and feasibility readiness. Future feasibility and pilot work should include primary-care clinicians, commissioners and senior budget holders; test the standard in services with different staffing, referral and booking arrangements; and incorporate descriptive workload and cost analyses, including survivor time-cost, staff time and shifts in demand between services, before wider adoption is considered.

Within these constraints, the study provides a mechanism‑grounded, equity‑focused accountability standard for post‑treatment survivorship transitions, with a traceable line from observed failures to minimal design features and feasibility indicators. The contribution is not evidence of effectiveness; it is a testable specification that makes ownership, timelines and closure visible, defines where triage, booking and escalation authority should sit, and identifies what should be monitored before wider implementation. If feasibility work shows that the standard can be applied reliably and equitably in routine care, it may offer a practical route away from reliance on survivor persistence and informal staff workarounds during post-treatment transitions.

## Methods

### Study design

We conducted a two‑stage sequential qualitative study (June–November 2025; Fig. S1) using a systems‑thinking lens and implementation frameworks to link observed accountability failures to design features and feasibility indicators.

Stage A mapped examined how handovers broke down at completion of active cancer treatment. Stage B used four accelerated experience‑based co‑design (AEBCD) workshops to translate those findings into a handover standard that could be tested in routine practice^71,72^. We focused on cancer survivorship because completion of active treatment is a high‑risk point with recurrent coordination demands. The standard was specified for empirical testing in survivorship services first, while allowing later adaptation to other post-treatment transitions.

### Setting, participants and sampling

Survivors were recruited through cancer charities and completed an online expression‑of‑interest form. The form confirmed eligibility, including adult status, tumour pathway, and time since treatment, and collected contact details, preferred contact method, availability, and interest in interview or workshop participation; it also invited optional demographic information, including gender, ethnicity, and postcode/general location. These data were used to monitor the emerging sample and support purposive recruitment for variation across tumour group, time since treatment, and relevant demographic or access characteristics. We did not use a formal quota matrix; instead, we reviewed the emerging sample iteratively during recruitment to identify underrepresented groups and guide subsequent invitations. We purposively sampled adults who were at least six months post‑treatment across three contrasting pathways: head and neck cancer (high complexity; functional late effects)^16^, colorectal cancer (high volume; established pathways; often primary‑care‑led follow‑up)^73^, and gastrointestinal stromal tumour (rare; long‑term specialised surveillance) ^74^.

Healthcare professionals were recruited from two London‑based NHS Trusts and third‑sector partners through direct invitation, existing professional networks, and limited colleague referral. We sampled for variation in role, service setting, and involvement in post-treatment survivorship handovers. The final sample included specialist medics, clinical nurse specialists, allied health professionals, psycho‑oncology practitioners and third‑sector staff.

Sampling was guided by the information power principle^75^, where adequacy was judged by the richness and relevance of the data in relation to the study aims rather than a fixed numerical target. Recruitment and analysis proceeded concurrently, and Stage-A recruitment ceased when additional interviews were no longer adding new mechanisms relevant to the study aims. For Stage-B we re‑invited Stage-A participants and used stratified convenience sampling to retain variation by role and tumour group ^76^.

### Data collection

All interviews and workshops were conducted remotely using Microsoft Teams or Zoom. Stage-A interviews comprised 65 (38 survivors; 27 professionals) semi-structured interviews lasting approximately 60 min. Interviews explored handover at completion of active cancer treatment, ownership of task and results, routes back into care, workarounds and the emotional labour involved in chasing test results, appointments and responses to queries.

Stage-B comprised four workshops with 21 returning participants (13 survivors; 8 professionals): two 90 min survivor workshops and two 60-min professional workshops. The survivor workshops developed a Patient Charter specifying what happens next, by whom and by when. The professional workshops examined feasibility, governance and the service architecture required to the proposed standard. About a week before the workshops, returning participants (20/21) completed a brief questionnaire prioritising transition challenges; aggregated responses were used to shape workshop agendas and to check resonance with Stage-A findings.

The lead author (PK), implementation scientist with more than 15 years’ qualitative experience, conducted all interviews and facilitated the workshops. Participation was voluntary and participants could withdraw at any time.

### Analysis and integration

Stage-A analysis began with inductive coding using the Framework Method (NVivo 15.2) to identify recurrent failure mechanisms. We then mapped determinants using the Consolidated Framework for Implementation Research (CFIR)^77^. Stage-B transcripts and co‑design artefacts were analysed using Normalisation Process Theory (NPT) to appraise the prospective workability of the proposed handover standard in routine practice ^78,79^.

We subsequently built a joint display (traceability matrix) that linked observed mechanisms, CFIR determinants, NPT workability signals, candidate implementation strategies (ERIC taxonomy)^80^, early implementation outcomes (Proctor framework)^81^, and an aligned audit‑ready indicator^20,21,61,66^. This matrix documented the linkage between each recurrent Stage-A failure mechanism and its corresponding Stage-B minimal design feature, together with mapped determinants and workability constructs, selected implementation strategies, expected implementation outcomes, and aligned feasibility indicators. These elements were assembled into an Implementation Research Logic Model to produce a programme theory and feasibility specification for the handover standard (Tables 3, S7 and Fig. S2) ^82^.

To make diagnosis-to-design traceability explicit, we then assembled a structured traceability matrix that linked each recurrent Stage-A failure mechanism to its corresponding Stage-B minimal design feature, mapped determinants and workability constructs, selected implementation strategies, expected implementation outcomes, and aligned feasibility indicators (Table S7). This matrix was used as an audit trail when checking consistency between Stage-A mechanisms, Stage-B design features, and the feasibility specification.

### Feasibility planning and measurement

We defined four equity‑stratified feasibility indicators aligned with the four components of the post‑treatment handover standard and labelled I1–I4 for traceability: time‑to‑acknowledgement (I1), loop‑closure within service timeframes (I2), results‑ownership compliance (I3) and channel‑concordance (I4). Operational definitions (start and stop rules, numerators and denominators, inclusion and exclusion criteria, missingness handling and equity strata) are summarised in Table 2 and detailed in Table S6. Indicators are specified as service level feasibility endpoints and will be reported descriptively as run or Statistical Process Control charts with denominators, equity stratification and a missingness row each cycle. The study is not powered for effectiveness and no inferential testing is planned. This aligns with a learning‑health‑system approach, in which routine process data feed back into iterative adjustment of pathways and thresholds at service level ^83^.

### Rigour and credibility

An implementation scientist and a public health specialist double coded 15% of transcripts; discrepancies were discussed and reconciled before final indexing. Analytic decisions and coding refinements were documented in an audit trail. Reporting follows SRQR and StaRI guidance ^84,85^.

### Reflexivity

The research team comprised an implementation scientist (PK), a public health specialist (BS) and clinical academics (DG, PL, AM, JG) working mainly in specialist care settings. This positionality provided contextual understanding of survivorship pathways but also risked normalising specialist-service boundaries and the amount of coordination work survivors are expected to carry. Structured debriefs and reflexive memos were used throughout the analysis to question whether familiar specialist practices were being treated as acceptable rather than scrutinised as system problems, these discussions were used to challenge interpretations that would have left ownership implicit, treated survivor chasing as routine, or assumed that digital routes were sufficient for follow-up. Input from the patient representative (KL‑S) was used to test these interpretations against survivor experience and to keep communication needs and non-digital access visible in the developing standard.

### Ethics and data management

Ethical approval for this study was granted by the East Midlands—Leicester South Research Ethics Committee (REC reference 25/EM/0132; IRAS project ID 356243), with Health Research Authority and Health and Care Research Wales approval for the study. The study was conducted in accordance with the Declaration of Helsinki. All participants provided written informed consent before participation. Audio-recordings were transcribed verbatim, pseudonymised and stored on secure institutional systems with restricted access under a registered Data Protection Impact Assessment. Participants were reimbursed at £60 per hour.

### Patient and public involvement (PPI)

A Patient Advisory Group contributed to protocol and materials, and a patient representative contributed to drafting and reviewing the manuscript. PPI activities are reported using GRIPP2.

## Supplementary information

Supplementary Materials (Final) v1.1

## Data Availability

The qualitative datasets generated and analysed during this study contain potentially identifiable information and were collected under REC/HRA approval (25/EM/0132). In line with these approvals, full transcripts are not publicly available to protect participant privacy. Anonymised excerpts and analytic materials may be available from the corresponding author on reasonable request, subject to a data-sharing agreement and relevant governance approvals.

## Code availability

No custom computer code or scripts were used to generate the results reported in this study.
