# Supplementary material for: The mirrored burden and accountable handovers in cancer survivorship: co‑designing a post‑treatment transition standard
Source: Npj Health Syst. 2026 Aug 5;3:67. doi: 10.1038/s44401-026-00125-9 (PMC13438100; doi:10.1038/s44401-026-00125-9)
Supplement: Supplementary file 1 [file 44401_2026_125_MOESM1_ESM.pdf]

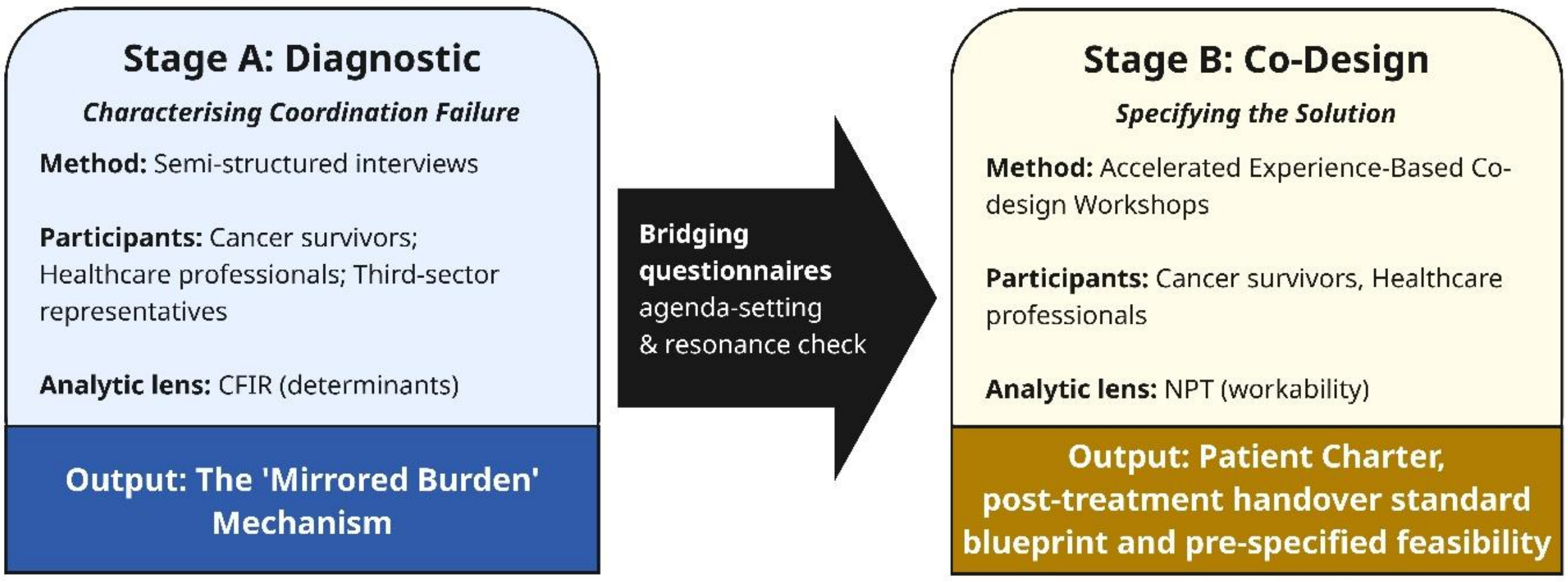

Figure S1. Two-stage sequential qualitative design and outputs (Stage-A diagnostic to Stage-B co-design). Stage-A interviews were analysed inductively to derive the 'mirrored burden' mechanism, with determinants subsequently mapped using CFIR. Brief bridging questionnaires informed Stage-B agendas and validated resonance with Stage-A themes (aggregate responses used for agenda setting, not formal analysis). Stage-B co-design (NPT-appraised workability) produced a survivor-defined Patient Charter, a specialist-facing blueprint, and pre-specified indicators.

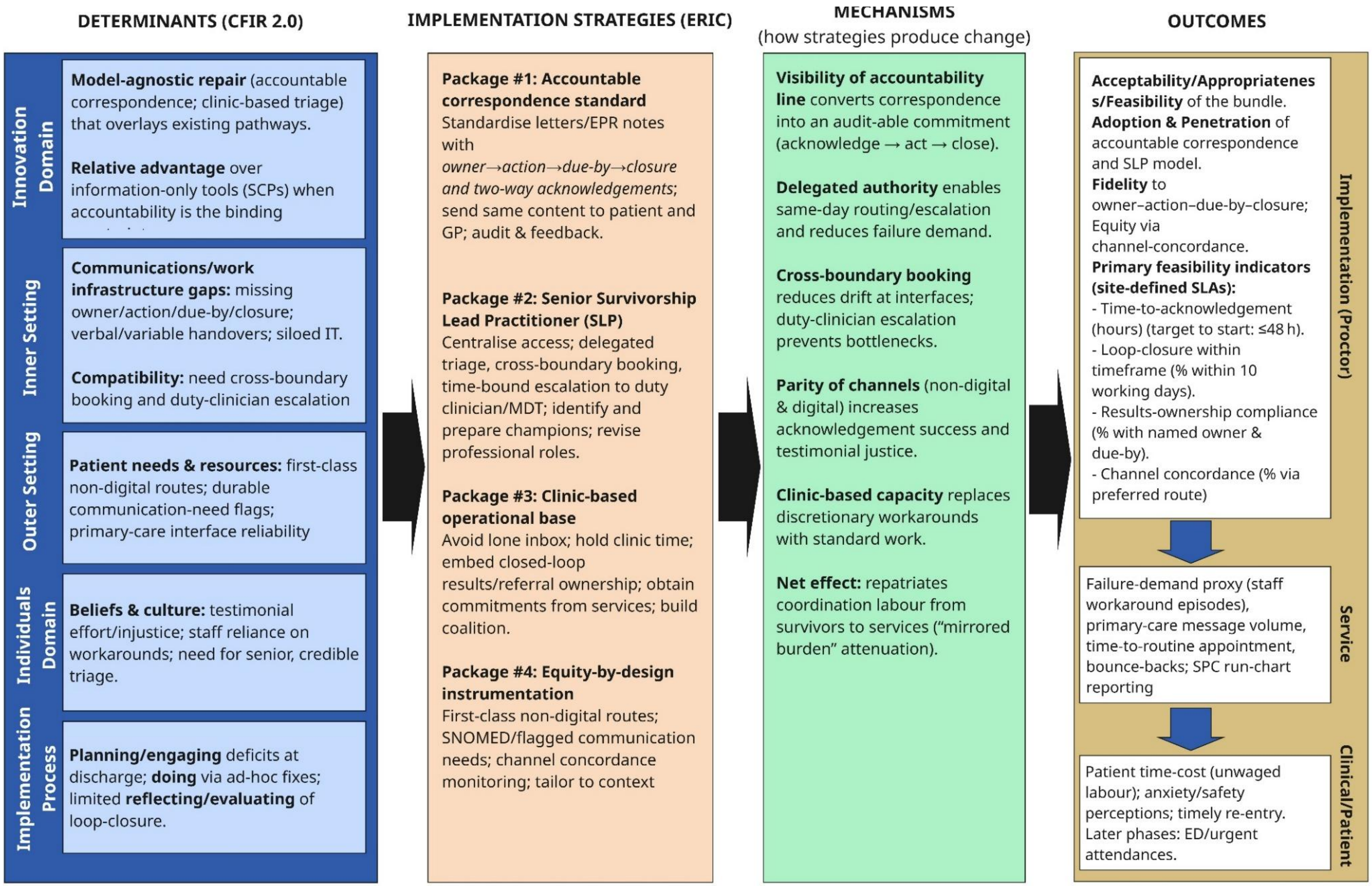

Figure S2. Implementation Research Logic Model: CFIR determinants from ERIC-aligned implementation packages for the post-treatment handover standard; these packages activate mechanisms (visibility of the accountability line, delegated authority, cross-boundary booking, parity of channels, clinic-based capacity) that in turn produce early implementation outcomes (acceptability, adoption, fidelity, feasibility, penetration, appropriateness, equity). The primary feasibility read-outs are the equity-stratified indicators (time-to-acknowledgement, loop-closure within timeframe, results-ownership compliance, channel-concordance) plotted as run or SPC charts with balance measures.

Table S1. Stage-A diagnostic joint display (mechanism, exemplar quotes and CFIR). Each row shows a transition failure that contributes to the mirrored burden, with paired survivor and HCP quotations to make the symmetry visible and a mapping to CFIR determinants.

Failure mechanism	Inductive anchor (what we observed repeatedly)	Representative survivor quotation	Representative professional quotation	CFIR 2.0 determinants
**1) Cliff-edge discharge and loss of named ownership.**	Abrupt end to treatment; uncertainty about "what happens next" and who owns next steps; impersonal letters instead of a structured handover.	"I've been through all that and then to just be released and told you're going to go for scans every whatever six months, whatever it was. That's it. Not really much more to it and it was probably just a letter as well that that told me that. It was kind of impersonal, I think." (P[09]-GIST-Survivor).	"I think the worry is that level of understanding and knowledge around their cancer, their treatment and their ongoing sort of post treatment challenges... There isn't a clear pathway and I think that's sort of one big barrier for people. There isn't always that go to phone number that they can pick up, which they probably had while they were on active sort of care, surveillance and treatment at that time." (HCP[17]-Third-sector).	Implementation Process-Planning; Implementation Process-Engaging; Inner Setting-Communications; Outer Setting-Patient Needs and Resources.
**2) Parallel coordination work**	Survivors chase letters/results because systems are fragmented; staff "bend rules" to push cases through.	"Sometimes I get asked, 'Oh, could you follow up with Charing Cross? Can you ask them to send letter?' I get that a lot. 'Can you ask and send your last clinic letter? Because we don't share the same systems?' Then as a patient, I have to go back, chase the secretary. 'Hey, they need you to send the clinic letter over.' So the patient is having to kind	"Often, we've tried various different strategies to support them as best we can already and to remain in a community setting, and it's not working. So that's why we feel that we need to take that approach, to bend the rules, so to speak, to get a bit more urgent support for them" (HCP[05]-AHP).	Inner Setting-Work Infrastructure; Inner Setting-Communications; Implementation Process-Doing; Innovation-Adaptability.

		of do a bit of that." (P[22]-HNC-Survivor).		
**3) Testimonial effort and epistemic inequity**	Survivors must repeatedly re-legitimise symptoms to convert acknowledgement into an action; clinicians recognise under-reporting and the need to prompt.	"I don't actually have to live like this, like, you need to help me… I was not rude, but I was very firm and I was like, I'm not leaving till I get help." (P[08]-GIST-Survivor).	"It feels very you have to be a very assertive patient that probably has English as your first language to get good access to the services that are out there… I think there are definitely vulnerable patient groups that miss out on that." (HCP[10]-Consultant).	Inner Setting-Culture: Learning-centredness; Human-Equality-centredness; Recipient-centredness; Outer Setting-Local Attitudes.
**4) Interface and preparation gaps**	Boundary failures and inconsistent preparation amplify risk; variable or purely verbal plans; survivors left to find information while recovering.	"It seems to have changed a couple of times. So, at first I was told you'll get PET scans every three months. Then I was told by someone else, "I know we wouldn't give you that much radiation. You'll have MRIs every three months". That didn't hasn't ever happened. I've just been scoped in the nasal cavity every three months. So, there doesn't seem to be a set, well, there wasn't a set care plan there." (P[22]-HNC-Survivor).	"Nine times out of ten patients are referred back… something basic that could have been dealt with in the community" (HCP[14]-CNS).	Outer Setting-Partnerships & Connections; Inner Setting-Communications; Implementation Process-Planning.
**5) Equity-relevant access barriers**	Digital and communication barriers make acknowledgement and re-entry unreliable; survivors	"With your reports and things like that, when you do get them, and even my consultant does this, they don't give it in layman's terms." (P[27]-HNC-Survivor).	"I think there is a presumption. I find it slightly baffling that everybody has a smartphone and assumption that everybody is digitally capable enough to be able to use that smartphone effectively to access all the	Outer Setting-Local Conditions; Inner Setting-Information Technology Infrastructure; Communications; Innovation-Design; Adaptability.

	request first-class non-digital routes.		things that we're expecting them to access. I don't always think it's generational" (HCP-[09]-AHP).	
**6) Buffering factors (negative cases)**	When a reachable named anchor exists, loops close and anxiety eases; "open door" policies help when clearly explained.	"He [oncologist] was just absolutely amazing through my treatment and since obviously stopping the treatment, just [given] incredible amount of support and help and phone calls and stuff. He gave me his number. We're still in contact now... We talked about recurrence, [and] things to look out for. He was always open to moving scans, blood tests or whatever, if needed," (P[06]-CRC-Survivor).	"I call it open door policy to my patients [...] the reason why I use the open door policy is because it's a picture, it's something in their head that shows that the door is always open to come back to me and when we say patient initiated follow up." (HCP[12]-AHP).	Inner Setting-Relational Connections; Inner Setting-Culture: Recipient-centredness; Implementation Process-Reflecting & Evaluating; Engaging.

Table S2. Questionnaire-prioritised agenda items (pre-workshop)

Cohort	Top-ranked priorities	Convergence with Stage-A	How used in workshops
**Survivors**	Discharge “void”; named owner; two-way acknowledgement; short, plain-language CTP; proactive mental-health check-ins; first-class non-digital routes; durable communication-need flags.	Cliff-edge discharge and loss of named care ownership; parallel coordination work; testimonial effort and epistemic inequity; equity-relevant access barriers.	Set Patient Charter baseline and parity rules; defined OADC and status visibility (“track it”).
**Professionals**	Overbooked/fragmented clinics; no cross-service booking; ambiguous results ownership; fragile interfaces; stress from workarounds; need a single front door led by a senior clinician.	Parallel coordination work; interface and preparation gaps; mirrored burden in coordination.	Specified SLP as clinical triage with delegated booking/escalation from a clinic base; default results ownership; re-entry service-level agreements.

Table S3. Patient Charter: survivor-specified service functions (operational definitions).

Domain	Function	Operational definition (what the service must do)
**Relational accountability**	Named clinical anchor & continuity	A named clinician (senior CNS/AHP) who knows the survivor's history and is directly reachable; continuity over time.
	Explicit authority & seniority	Anchor is clinically credible and empowered to triage, refer, book across services, prescribe (where credentialled), and escalate with deadlines; not a "lone inbox".
**Infrastructural accountability**	Care Transition Plan	Short, action-oriented handover at completion of active treatment (what happens next, by whom, by when), first check-in booked; the same plan to survivor and healthcare professionals.
	Closed-loop documentation and acknowledgements (OADC visible)	Every result/task shows OADC; two-way acknowledgements; identical content to survivor and healthcare professionals; visible status ("where my query is").
	Profile/Pack + durable alerts	A short "profile" (history, late-effects risks, communication needs) with visible alerts so survivors do not have to retell their story; surfaces before the consult.
	Self-referral / re-entry "for life"	A simple, guaranteed route back into specialist input beyond five years; requests triaged promptly against time-bound targets.
	One-stop digital hub with human backup	Single place to view status, message the team and book; integrated with records; first-class phone/letter alternatives for those not using apps.
**Designed equity**	Accessibility & parity (non-digital routes; communication-need flags)	Non-digital channels (phone/letter/relay) are first-class; durable communication-need flags propagate across services; channel of choice honoured; report channel-concordance.
	Trauma-sensitive psychosocial check-ins	Proactive, normalised mental-health safety-netting, with red-flag triggers that escalate to appropriate services.
	Information parity & rights ("Is this normal?" channel)	Plain-language information on services and legal/work rights; a clinically overseen "Is this normal?" triage channel; not limited to one Trust.
	Peer mentorship (with safeguards)	Trained, supervised peers complement clinicians; accuracy/boundaries/escalation rules; options for age-specific groups.
	Relational governance & patient voice (Patient Council)	A standing Patient Council able to trigger service review (e.g., adapted patient-safety methods).

Note: Functions in this Charter are synthesised into the four-component post-treatment handover standard in Table 2 (accountable correspondence, clinic-based SLP, clinic-based resolution capacity and equity safeguards). Trauma-sensitive check-ins draw on established training such as #CheckWithMeFirst;[1] channels remain clinically overseen.

Table S4. Service architecture and workability of the post-treatment handover standard (NPT-aligned). Operational specification of the Survivorship Lead Practitioner model with delegated authority and interfaces. Right-hand columns map each element to Normalisation Process Theory: Coherence (what it is), Cognitive Participation (who buys in), Collective Action (what makes it work), and Reflexive Monitoring (how staff will judge it). Reliability is tracked as run/SPC charts with equity stratification and a missingness row; thresholds carry version IDs.

**Element**	**What it is** (operational)	**Delegated authority** (within scope)	**Interfaces and boundaries** (delineation)	***Normalisation Process Theory (NPT) Constructs***
**Profile and seniority**	Senior CNS/AHP named as the survivor-facing anchor (Survivorship Lead Practitioner, SLP) with a visible contact route.	Recognised at Trust/ICS level; can request action with due-by dates; triage and escalate.	Complements CNS and Macmillan navigator roles; does not replace surveillance or MDT decisions.	• **Coherence (differentiation, communal specification):** the SLP is understood as a clinical triage role with booking and escalation authority, not a logistics helpline, and directorates share a working aim of faster closure, fewer repeat contacts, and safer escalation.<br>• **Cognitive participation (initiation, legitimation):** sponsors create and resource a senior CNS/AHP post, and the credentialled status of the role makes cross-service action appropriate and expected.<br>• **Collective action (skill-set workability, contextual integration):** triage criteria and escalation algorithms are explicit and rehearsed; protected clinic time, cross-boundary booking rights, shared-care record visibility, and information-sharing agreements are in place.<br>• **Reflexive monitoring (systematization, reconfiguration):** teams track time to acknowledgement, loop closure, results ownership, and channel concordance as run/SPC charts with equity splits, then version-log and adjust SOPs and thresholds in response.
**Clinical triage**	First clinical point of contact; judges urgency; routes to self-care, community, or acute pathways.	Bring-forward; initiate protocolised diagnostics; escalate to duty clinician/MDT.	Uses existing pathways with cross-boundary booking rights and named primary-care contacts; letters/EPR notes carry the OADC; time-bound	• **Coherence (differentiation, individual specification):** triage is recognised as clinical decision-making rather than advice-only navigation; staff know who triages, who books, who escalates, and who closes.<br>• **Cognitive participation (enrolment, activation):** diagnostics, booking teams, and admin hubs commit to live scheduling access, and escalation drills and rota rehearsals keep procedures active.

			escalation functions to a duty clinician/MDT when capacity blocks closure.	• **Collective action (interactional workability, relational integration):** booking occurs at first contact where feasible and OADC is recorded in notes and letters; tumour teams accept SLP triage decisions without duplicating work.<br>• **Reflexive monitoring (systematization, reconfiguration):** triage outcomes and safety events are recorded with subgroup reporting; criteria and timers are tightened when SPC charts show delay, rework, or safety concerns.
**Visibility and role clarity**	Role card and service specification circulated across oncology, diagnostics, admin hubs, and primary care.	Right to return items lacking owner/action/due-by/closure; right to two-way acknowledgements.	Standard letters and EPR notes use the OADC; Directory lists named healthcare professionals contacts; acknowledgement/closure status is visible across oncology and primary care.	• **Coherence (individual specification, internalization):** templates and role cards make acknowledgement, closure, and returns responsibilities explicit, and staff can see owner, action, due-by date, and status for each item.<br>• **Cognitive participation (initiation, enrolment):** clinical leads endorse the role specification and OADC templates; a directory lists named contacts in oncology and primary care with agreed response timeframes.<br>• **Collective action (interactional workability, contextual integration):** OADC appears on all survivorship-relevant correspondence and EPR notes, and digital tools display acknowledgement and closure status across oncology and primary care.<br>• **Reflexive monitoring (systematization, communal appraisal):** acknowledgement times and closed-loop rates are charted with a missingness row; cross-service huddles review late or returned items and agree corrective actions.
**Embedding in standard care**	Single front door for survivorship queries (phone, text, email, relay); non-digital parity.	Trigger re-entry; book across clinics; initiate rehabilitation and psychosocial referrals.	Aligned to scheduled follow-up and patient-initiated follow-up; parity rules for non-digital routes; durable communication-need flags;	• **Coherence (differentiation, communal specification):** the “front door” is framed as integrated care rather than an app-only portal, with shared aims of clear re-entry, fewer repeat contacts, and reliable closure.<br>• **Cognitive participation (enrolment, activation):** clinics and admin hubs sign up to channel-specific response times; rota maintenance and dashboard surveillance keep the front door visible and staffed.

			re-entry/escalation SOPs set response times across the primary-specialist boundary.	• **Collective action (interactional workability, contextual integration):** contacts flow across phone, text, email, and relay with durable communication-need flags; resources are allocated to maintain parity for non-digital routes alongside digital tools.<br>• **Reflexive monitoring (systematization, reconfiguration):** channel-concordance is monitored by subgroup; routing rules and capacity are revised where equity gaps or high abandonment rates appear.
**Operational base and resolution capacity**	Staffed clinic base that holds time and relationships; not a stand-alone inbox.	Run group sessions; coordinate follow-ups; escalate resource constraints to managers.	Avoid new parallel queues; escalate early when capacity blocks closure.	• **Coherence (differentiation, internalization):** the service is understood as a clinic-based problem-solving function, not a message relay, and staff judge it credible, reachable, and timely.<br>• **Cognitive participation (legitimation, activation):** seniority and job plans confer authority to act across organisational boundaries; standing meetings and dashboards keep capacity, queues, and escalation routes in view.<br>• **Collective action (contextual integration, interactional workability):** booking rights, clinic time, funding streams, and duty clinician/MDT escalation are specified; live booking and documented closure occur within agreed timeframes.<br>• **Reflexive monitoring (systematization, communal appraisal):** time to closure and aged queues are tracked with balance measures (for example primary-care message volume and time to routine appointment); capacity reviews adjust workloads, thresholds, and escalation rules.

Notes: Banding expectations, escalation, and rejection of a lone-inbox derive from workshop testimony and worksheet entries. ***Abbreviations:*** AHP (allied health professional); CNS (clinical nurse specialist); CTP (Care Transition Plan); EPR (Electronic Patient Record); GP (general practitioner); ICS (Integrated Care System); MDT (multidisciplinary team); SLA (service-level agreement); SLP (Survivorship Lead Practitioner)

Table S5. Full count-as-implemented gates

Post-treatment handover standard component	Gates for the post-treatment handover standard
**1. Accountable correspondence (OADC)**	• Trust-approved OADC templates active in EPR and clinic letters (owner, action, due-by, closure fields).<br>• Two-way acknowledgement rule defined and operational (read-receipt or manual audit log).<br>• Identical content automatically sent to survivor and GP (or documented operational workaround).<br>• Status field visible to teams (acknowledged/in-progress/closed) across oncology-primary care.<br>• Right-to-return non-OADC items agreed and enacted (SOP + escalation).
**2. Clinically senior SLP**	• Post banding and seniority specified; job plan with protected clinic time.<br>• Written delegated authority (triage criteria; booking rights; escalation to duty clinician/MDT with timers).<br>• Single “front-door” contact channel(s) published; not a generic admin helpline.<br>• Directory of named HCP contacts with response SLAs.
**3. Clinic-based resolution capacity**	• Named clinic base with rota; coverage for absence.<br>• Live access to cross-service booking/scheduling and diagnostics.<br>• Bring-forward lists and aged-queue review routine.<br>• No parallel “shadow queues”; inboxes monitored with published SLAs.
**4. Equity safeguards**	• First-class non-digital routes (phone/letter/relay) resourced and monitored.<br>• Durable AIS/SNOMED communication-need flags implemented; propagated across referrals/letters.<br>• Preferred channel recorded at each contact; channel-concordance reported with equity splits and a missingness row.

Note: Gates are pre-specified conditions for future feasibility evaluation of the four-component post-treatment handover standard in Table 2. They define the minimum service-level conditions used to judge whether a component is in place for feasibility testing; site-defined timeframes and thresholds remain subject to local calibration and version control.

Table S6. Indicator dictionary (full operational definitions)

Indicator	Start → Stop rule (operational)	Unit	Numerator / Denominator	Inclusions / Exclusions	Missingness handling	Equity strata (report every cycle)	Display & thresholds	Balance measures (tracked in parallel)	Primary data sources	Multiplicity / Notes
**Time-to-acknowledgement (I1)**	Start: timestamp when a survivorship contact reaches the service (phone call logged; message received; letter scanned/logged). Stop: first two-way acknowledgement documented in EPR or audit log.	Hours (plus % within SLA)	# requests acknowledged within the site-defined SLA / All requests	Include all survivorship-relevant contacts; exclude auto-notifications without two-way contact; de-duplicate repeat contacts within 24 h (document rule).	Code start/stop missing separately with reason; retain in denominator; show missingness row.	Language; Disability (AIS); Ethnicity; IMD quintile; Age; Tumour group.	Monthly run/SPC; version-logged threshold (e.g., ≤ 72 h). Denominators shown.	Primary-care message volume; Time-to-routine appointment.	EPR contact/comm logs; shared mailbox/telephony logs; manual acknowledgement log if needed.	Pre-specified feasibility indicator for future feasibility evaluation; descriptive reporting only. Not powered or intended as an effectiveness outcome.
**Loop-closure within timeframe (I2)**	Start: request logged/assigned with named owner. Stop: action completed and closure communicated per OADC Closure rule.	Percent	# actionable requests closed within timeframe / All actionable requests	Exclude "informational only" requests by pre-rule; classify and document.	Code "no closure/unclear stop" with reason; retain in denominator; show missingness row.	Same as I1.	Monthly run/SPC; version-logged timeframe by action type; denominators shown.	Aged open-queue count; Re-contact within 14/30 days.	Task/queue registers; letter/EPR closure notes; escalation logs.	Pre-specified feasibility indicator for future feasibility evaluation; descriptive reporting only. Not

										powered or intended as an effectiveness outcome.
**Results-ownership compliance (I3)**	For each reportable result ordered in survivorship: OADC fields Owner, Due-by, Closure note are present and visible to survivor and GP.	Percent	# reportable results with all three fields present & visible / All reportable results	Include imaging, pathology and key labs marked as “reportable”; composite orders counted per reportable element.	Field-level coding of what is missing (owner/due-by/closure/visibility); retain in denominator; show missingness row.	Same as I1.	Monthly run/SPC; show component-level non-compliance in footnotes if needed.	Time from result available → closure; rate of “owner reassignments”.	Order comms module; clinic letters; GP-copy status/Shared Care Record.	Pre-specified feasibility indicator for future feasibility evaluation; descriptive reporting only. Not powered or intended as an effectiveness outcome.
**Channel-concordance (I4)**	An interaction is concordant if initiated and acknowledged via the documented preferred channel (phone, letter, relay, email/app).	Percent	# interactions via recorded preferred route / All interactions	Include all service contacts; exclude automated bulk notices.	Code “preference unknown”; retain in denominator; show missingness row.	Same as I1.	Monthly run/SPC with equity splits; show parity check (digital vs non-digital SLAs) in caption.	Difference in SLA by channel; abandonment rate when routed to non-preferred channel.	EPR preference field; comms logs; AIS flags; directory/SLA logs.	Pre-specified feasibility indicator for future feasibility evaluation; descriptive reporting

	Non-digital routes must have parity.		only. Not powered or intended as an effectiveness outcome.

Reporting rules (apply to all four indicators): (i) display as run/SPC with version-logged thresholds; (ii) show denominators and a missingness row; (iii) stratify by the equity variables listed; (iv) co-plot balance measures. Indicators are pre-specified feasibility indicators for future feasibility evaluation; no indicator data were collected in this co-design study

Table S7. Full diagnosis-to-design traceability for the post-treatment handover standard from Stage-A failure mechanisms to Stage-B minimal features with enabling strategies and early outcomes. Each row links a Stage-A transition failure contributing to the mirrored burden to its Stage-B minimal design feature, the underlying determinants and workability (CFIR 2.0 and Normalisation Process Theory), selected ERIC implementation strategies and their local operationalisation, expected implementation outcomes (Proctor) and the aligned audit-ready indicator(s) from Table 2. Rows are conceptual, not statistical, and are therefore not subject to multiplicity correction. Indicators are pre-specified feasibility indicator for future evaluation of the post-treatment handover standard; this co-design study did not collect indicator data.

Mechanism (Stage-A)	Minimal Design Feature (Stage-B)	Determinants & Workability (CFIR 2.0; NPT)	Implementation strategies (ERIC; local operationalisation)	Implementation outcomes (Proctor)	Audit-ready indicator(s)
**Cliff-edge discharge and loss of named ownership**<br><br>Abrupt end to treatment; no visible owner, action, due-by date or closure rule; impersonal letters instead of a structured handover ("cliff-edge discharge").	Accountable correspondence in routine letters/EPR notes: OADC (Owner, Action, Due-by, Closure) fields, two-way acknowledgements, identical content to survivor and GP, cross-boundary booking and time-bound escalation.	**CFIR**: Implementation Process - Planning; Implementation Process - Engaging; Inner Setting - communications; Outer Setting - Patient Needs & Resources.<br><br>**NPT**: Coherence - Differentiation; Collective Action - Interactional workability; Reflexive Monitoring - Systematization.<br><br>**Operational signal:** OADC templates in routine use; right to return	• **Develop a formal implementation blueprint:** produce an OADC standard with letter/EPR templates, acknowledgement and escalation rules.<br>• **Change record systems:** build OADC letter and note templates; add mandatory OADC fields; provide a manual audit log where read-receipts are unavailable.<br>• **Conduct educational meetings and educational outreach visits:** short sessions for consultants, CNS/AHP and admin teams on using OADC and returning non-OADC items.<br>• **Obtain formal commitments:** directorate-level agreement that non-OADC correspondence can be returned for completion.<br>• **Audit and provide feedback:** monthly run/SPC charts on OADC completeness, time-to-acknowledgement and results-ownership compliance, stratified for equity.	Acceptability; Adoption; Fidelity	time-to-acknowledgement (I1); results-ownership compliance (I3).

		non-OADC items; status (acknowledged/in-progress/closed) visible across oncology and primary care.			
**Parallel coordination work ("patient as case-manager")**<br><br>Survivors must act as their own case managers, chasing letters and results across fragmented systems; staff "bend rules" and create informal workarounds when pathways stall.	Senior Survivorship Lead Practitioner (SLP) (senior nurse/AHP, advanced practice feasible) as first clinical triage from a clinic base, with delegated authority, cross-service booking and duty-clinician escalation.	**CFIR**: Inner Setting - Compatibility; Inner Setting - Access to Knowledge & Information; Inner Setting - Work Infrastructure; Implementation Process - Doing.<br><br>**NPT**: Coherence - Differentiation; Collective Action - Contextual integration; Reflexive Monitoring - Systematization.<br><br>**Operational signal:** Protected clinic time; cross-boundary booking rights; named escalation routes; default	• **Revise professional roles:** specify SLP tasks and delegated authority (triage, booking, escalation) in job plans and SOPs.<br>• **Create new clinical teams:** commission a clinic-based SLP service with protected sessions and advice lines rather than relying on informal CNS work.<br>• **Change record systems:** enable cross-boundary booking and default results-ownership assignment in the EPR.<br>• **Build a coalition:** identify surgical, oncology, diagnostics and admin leads who co-own booking and escalation flows.<br>• **Organize clinician implementation team meetings:** regular SLP-MDT huddles to review misroutes and queue risks.<br>• **Provide ongoing consultation:** early-phase (e.g. first 8 weeks) weekly review of triage/escalation decisions to refine criteria.	Adoption; Penetration; Feasibility	loop-closure within timeframe (I2).

		results ownership visible in the record.			
**Testimonial effort and epistemic inequity**<br><br>Survivors must repeatedly re-legitimise symptoms to convert acknowledgement into action; access favours assertive, English-speaking survivors, creating testimonial effort and epistemic inequity.	"Acknowledge → Act" rule in SOPs plus durable AIS-compliant communication-need flags and channel-of-choice recording; OADC fields used in plain-language letters.	**CFIR**: Inner Setting - Culture (learning-centredness; human-equality-centredness; recipient-centredness); Individuals - Capability; Individuals - Motivation; Outer Setting - Local Attitudes.<br><br>**NPT**: Collective Action - Relational integration; Cognitive Participation - Legitimation; Reflexive Monitoring - Communal appraisal.<br><br>**Operational signal:** Plain-language OADC in correspondence; visible communication-need flags	• **Identify and prepare champions:** two senior clinicians model "acknowledge then act" behaviour and OADC adherence.<br>• **Tailor strategies**: implement AIS-compliant communication-need flags and require a recorded preferred channel for all contacts.<br>• **Use advisory boards and workgroups:** PPI group reviews letters for OADC completeness and intelligibility.<br>• **Conduct local consensus discussions:** teams agree what constitutes acceptable testimonial effort and response times.<br>• **Obtain formal commitments:** services sign up to response SLAs (acknowledgement and closure).<br>• **Audit and provide feedback:** monthly review of acknowledgement delays, non-acted acknowledgements and returned non-OADC correspondence by equity subgroup.	Acceptability; Appropriateness; Equity; Feasibility	time-to-acknowledgement (I1, equity-stratified); loop-closure within timeframe (I2, equity-stratified)

		travelling across referrals and letters; service-level rules that acknowledgement is followed by timely action or explicit closure.			
**Interface and preparation gaps (verbal / variable handovers)**<br><br>Boundary failures and verbal or variable plans across sites; survivors left to "find" information while recovering; no consistent written care plan or pre-booked next step.	Short Care Transition Plan at completion of active treatment: concise "what / by whom / by when"; first check-in booked; identical content to survivor and healthcare professionals; cross-boundary booking and time-bound escalation.	**CFIR**: Outer Setting-Partnerships & connections; Inner Setting-Communications; Implementation process-Planning/Doing.<br><br>**NPT**: Coherence - Communal specification; Collective Action - Interactional workability; Reflexive Monitoring - Systematization.<br><br>**Operational signal:** Care Transition Plan completed at discharge, first check-in pre-booked, and the same plan	• **Develop a formal implementation blueprint**: integrate the Care Transition Plan into the wider survivorship standard and local pathways.<br>• **Develop educational materials**: concise CTP templates and guidance for clinicians on completing "what / by whom / by when".<br>• **Facilitate relay of clinical data to providers**: automatic sending of identical CTP content to survivor and relevant healthcare professionals.<br>• **Develop resource sharing agreements**: document cross-boundary booking rights and escalation routes with named contacts.<br>• **Conduct local consensus discussions**: agree pathway-specific due-by targets and escalation triggers for common actions.<br>• **Conduct educational outreach visits**: targeted support for clinics with high rates of open loops or incomplete CTPs.	Fidelity; Feasibility; Sustainability	Primarily: loop-closure within timeframe (I2); Secondarily: time-to-acknowledgement for post-CTP contacts (I1).

		visible in survivor and provider records.			
**Equity-relevant access barriers (digital/speech/ language)**<br><br>Digital and communication barriers make acknowledgement and re-entry unreliable; survivors explicitly request first-class non-digital routes and durable communication-need flags.	Parity by design: first-class non-digital routes (phone/letter/relay); SNOMED/AIS-coded communication-need flags; documented channel of choice that is honoured in practice.	**CFIR**: Outer Setting-Patient Needs & Resources; Outer Setting-Local Conditions; Inner Setting-Information Technology Infrastructure; Inner Setting-Access to Knowledge & Information; Innovation-Adaptability.<br><br>**NPT**: Collective Action - Contextual integration; Reflexive Monitoring - Systematization; Reflexive Monitoring - Reconfiguration.<br><br>**Operational signal**: Preferred channel recorded and used; parity rules applied so non-digital routes	• **Tailor strategies:** explicitly design first-class non-digital routes and parity rules; adapt protocols for hearing/speech/language needs.<br>• **Change record systems:** implement durable AIS/SNOMED communication-need flags that propagate across referrals and letters.<br>• **Promote network weaving:** maintain a directory of named contacts and SLAs across oncology, diagnostics and primary care.<br>• **Prepare patients/consumers to be active participants:** provide a one-page "how to re-enter care" sheet, including phone, relay and letter options.<br>• **Capture and share local knowledge:** quarterly review of channel-concordance and equity gaps; adjust routing rules and resourcing where disparities persist.	Appropriateness; Equity; Reach; Feasibility; Adoption.	Channel-concordance (equity-visible), (I4).

		have comparable response times and visibility.			
**Buffering factors (negative cases: reachable anchor & “open-door” policy)**<br><br>Where a reachable named anchor and clear “open-door” policy exist, loops close and anxiety eases; survivors do not experience the full mirrored burden.	Corollary of the SLP and Care Transition Plan features: a named, reachable anchor with authority and clear re-entry routes, framed as patient-initiated follow-up (PIFU) with explicit clinico-administrative closure rules.	**CFIR:** Inner Setting - Relational Connections; Inner Setting - Culture (recipient-centredness); Implementation Process - Engaging; Reflecting & Evaluating.<br><br>**NPT:** Cognitive Participation - Legitimation; Collective Action - Relational/Interactional workability; Reflexive Monitoring - Systematization.<br><br>**Operational signal:** Survivors know who to contact and how; re-entry contacts follow OADC and closure rules rather than	• **Revise professional roles:** embed the “open-door” function in the SLP role description and SOPs, with clear authority to act.<br>• **Build a coalition:** align MDT, primary care and diagnostics around the role of the named anchor and agreed re-entry expectations.<br>• **Change record systems:** ensure re-entry contacts via the anchor are visible, OADC-tagged and shareable across services.<br>• **Provide ongoing consultation:** use early re-entry cases to refine criteria and escalation rules, with regular debriefs.	Acceptability; Fidelity.	loop-closure within timeframe for re-entry contacts (I2); time-to-acknowledgement for contacts via the named anchor (I1).

	relying on<br>informal personal<br>favours.	

1. The Survivors Trust. About #CheckWithMeFirst [cited 2025 25 September]. Available from: https://thesurvivorstrust.org/training/checkwithmefirst/ accessed 25 September 2025.
2. Riley S, Burhouse A, Nicholas T. National Health Service (NHS) trust boards adopt statistical process control reporting: the impact of the Making Data Count Training Programme. *BMJ Leader* 2021:leader-2020-000357.
